# Philadelphia Correspondence

**Published:** 1868-09-01

**Authors:** 


### *PHILADELPHIA CORRESPONDENCE.*

PHILADELPHIA, *August* 3, 1868.

MEDICAL EDUCATION.—It is remarkable that in the discussions which have been maintained in the meetings of the American Medical Association on the above topic, men who have been supposed to be among the "*soundest* of the *sound*," should advocate the resolution which was offered two years ago relative to this matter. That the standard of medical education and profession should be high; that men who are to take lives in their hands should be amply qualified thus to do, no sensible man doubts or disputes; but that any man should be allowed to run on in any path he desires while *preparing* for his lectures; that he should be sent to our colleges unable to tell where Peru or Greenland are; unable, scarcely, to write his name properly, or certainly to spell with correctness. In short, devoid of a common school education; and in this state of mind thrust upon our colleges, and as he approaches the Dean is told, "Now, sir, you are here, and here you must stay four years. You must learn geology, botany, astronomy and philosophy, in addition to your regular studies of the profession." How absurd does this seem to us! Not that we have a lower opinion of medicine than we ought, or than others. Not because we think the physician goes well enough prepared to go from his college, and simply because it is a piece of injustice to the faculty and to the student. That many an one goes away from our colleges ill prepared for his duties, we grant; but is this the way to remedy it? We think not. On the contrary, it is just the way to check the prosperity of our colleges, and to dishearten many an one who, if properly instructed, might adorn his profession. Such a plan existing, and what would be the result? A man coming from some country village to Jefferson College, or Rush College, or elsewhere. He has been under his *preceptor* (?) a certain length of time; knows what the spinal column is composed of; knows, perhaps, the description of the *sphenoid* bone *by heart;* can tell you where the *glutæus muscles*, or the *sphnicter ani* are, but when told that he has to learn all about geology, etc., etc., and that he must stay "three years at the least," he holds up his hands in holy horror, and leaves for the plough again.

Now, what is the way to bring up the standard of medical education? How shall the colleges send forth men better prepared for their duties? Not in this way. Let the standard be raised in the *preceptor's* office. Here is the place, and no where else. Let him there go through the proper preparatory studies, and, if necessary, undergo an examination prior to entering the college. By such means medical gentlemen will go out into the world better fitted, and the colleges will be prosperous in the extreme. If such men as the "gentleman from Cincinnati," or the "tall man with gray hair," from Philadelphia would advance such an idea as this in regard to medical education, and not such Utopian ones as they did, they could do far more good to the world, and reflect far more credit on themselves, with the hope of *sometime* seeing their scheme successful. Yours,

